# Design and Implementation of Sound Searching Robots in Wireless Sensor Networks

**DOI:** 10.3390/s16091550

**Published:** 2016-09-21

**Authors:** Lianfu Han, Zhengguang Shen, Changfeng Fu, Chao Liu

**Affiliations:** 1 School of Electronic Science, Northeast Petroleum University, Daqing 163318, China; wxc0818@gmail.com (C.F.); wangxc210@nenu.edu.cn (C.L.); 2 Beijing Institute of Automatic Control Equipment, Beijing 100076, China; szg0818@gmail.com

**Keywords:** wireless sensor network (WSN), microphone array, magneto-resistive sensor, sound recognition and localization, target searching, robot system

## Abstract

A sound target-searching robot system which includes a 4-channel microphone array for sound collection, magneto-resistive sensor for declination measurement, and a wireless sensor networks (WSN) for exchanging information is described. It has an embedded sound signal enhancement, recognition and location method, and a sound searching strategy based on a digital signal processor (DSP). As the wireless network nodes, three robots comprise the WSN a personal computer (PC) in order to search the three different sound targets in task-oriented collaboration. The improved spectral subtraction method is used for noise reduction. As the feature of audio signal, Mel-frequency cepstral coefficient (MFCC) is extracted. Based on the K-nearest neighbor classification method, we match the trained feature template to recognize sound signal type. This paper utilizes the improved generalized cross correlation method to estimate time delay of arrival (TDOA), and then employs spherical-interpolation for sound location according to the TDOA and the geometrical position of the microphone array. A new mapping has been proposed to direct the motor to search sound targets flexibly. As the sink node, the PC receives and displays the result processed in the WSN, and it also has the ultimate power to make decision on the received results in order to improve their accuracy. The experiment results show that the designed three-robot system implements sound target searching function without collisions and performs well.

## 1. Introduction

Some research studies which center on visual human machine interaction have been performed [1,2,3,4]. However, the robotic audition system that integrates the multifunctional task of recognizing, localizing and searching sound targets plays much a more important role in darkness or shaded areas and all-direction detection conditions [5,6]. It is said that such robots in real environments have not been commonly developed although cooperative multi-robot systems through wireless sensor networks (WSNs), using the robot’s audition capabilities have been studied by various research laboratories [7].

Animals have evolved more sophisticated strategies to address the difficulty of searching for sound sources, and the most significant feature lies in the complex structure of the ear’s audio system. How to simplify its construction is also studied by various research laboratories, but few achievements have been made because the animal’s ear audio system itself is rather complex. Therefore, the microphone array structures used in research are generally huge, for example, a 128-channel huge microphone array was used for sound source localization in [7]. It is unrealistic that such structure be applied to a robot in a WSN. Furthermore, searching for a sound target is not a straightforward task for a robotic agent, and it also involves sound identification, localization and motor driving. Therefore, we aim to implement such a robot system in task-oriented collaboration (TOC) using wireless senor networks for searching sound target, which also can simplify its structure and improve the accuracy.

A sound target searching robot system using the WSN technology represents a new research field for the application of robots. It is significant in improving the ability of decision-making for sound target searching and it also integrates the multiple-sensor technology, pattern recognition, wireless sensor networks and robot technology. The advantage of our research is to implement such an instrument that the robot system searches and search cooperatively for a sound source by WSN, meanwhile, we also apply the microphone sensor array [8,9,10,11,12] which is used for sound recognition and localization, magnetic sensor technology [13,14,15], for target searching and wireless sensor network communications [16,17,18] for exchanging information among multiple robots and PC. It aims mainly to accomplish the searching target in TOC, which fits for the development trends of intelligent robots. The robot system is useful in the dangerous operations, such as fire protection and rescue action [19,20]. Furthermore, the audio robot system can play an important role in home care and invisible conditions, and different applications can also be found in multimedia teleconferencing systems and hearing aids.

This paper describes in detail the structure of a three-robot system implemented in a WSN, and its sound recognition, sound localization and sound target searching algorithm. The main feature is its simple physical microphone array system framework consisting of a four microphone planar array, while it has the following advantages: firstly, it combines sound identification with improved location technology. Then, the robots search for a sound target in a cooperative way. Finally, the robots perform their task without collisions using WSN technology. In contrast, the previous research was based largely on huge numbers of microphone sensors, and the sound recognition was also isolated from sound localization, and it fulfilled its task by itself without TOC. The three-robot system has taken appropriate actions to accomplish the searching task, and thus a significant improvement in the functional implementation has been achieved.

This research includes three robot vehicles with simple construction; it also focuses on the new DSP TMS320F28335 processor to achieve the purpose of searching three respective sound sources. The three robots coordinate with each other in the WSN, which can optimize and simplify the structure to accomplish the same task. To sum up, a system that centers on various sensor measuring technology and applies an improved audio pattern recognition algorithm to the robot area based on the DSP processor has been implemented.

## 2. Structure of Robot System in WSN 

**Figure 1:**
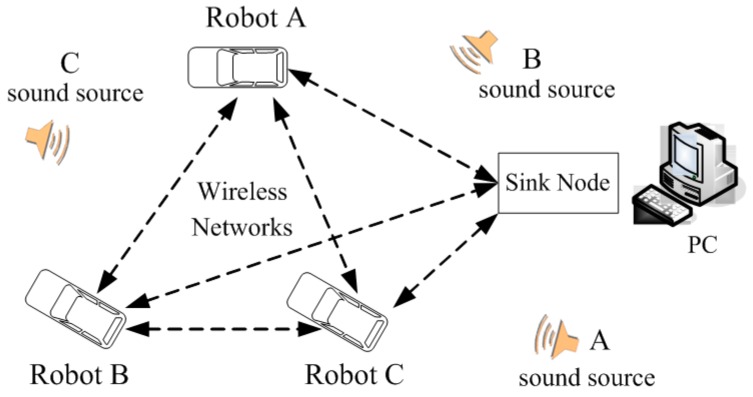
Architecture of three-robot system in WSN.

Figure 1 shows the architecture of the three-robot system in a WSN. It consists of three robots as the WSN nodes, a PC as sink node and three different sound source targets.

**Figure 2:**
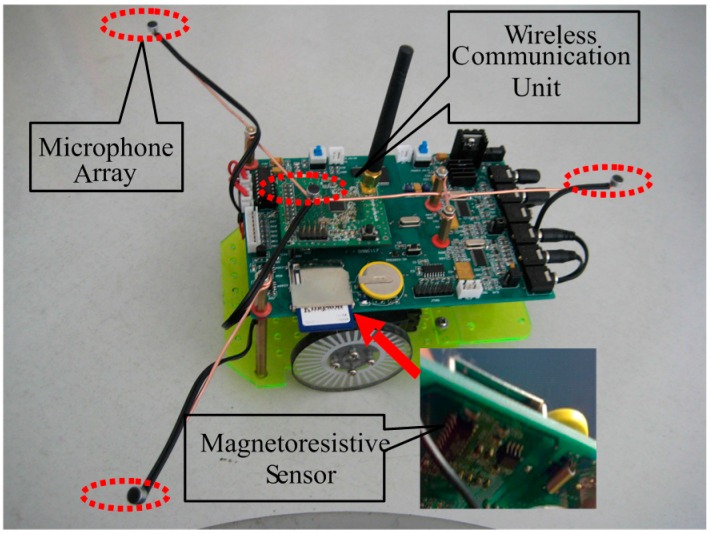
Photograph of a mobile robot.

Based on the application of sensor technology, each robot identifies and locates its corresponding sound source target. As the sink node in the WSN, the PC in Figure 1 is in a fixed position, and receives self-defining coded data information on the current heading angle, received signal strength indicator (RSSI), the result of sound identification and sound source position from the every robot node in detail. To improve the accuracy of sound recognition, the PC has decision-making power to give an order, and determines the merging result of each item, which guides the right robot to search the right sound source target effectively. Due to the use of WSN, the robots can accomplish their task in collaboration. As a wireless sensor network node, each robot itself is composed of 4-channel microphone array, magneto-resistive sensor, new DSP processor and the wireless communication unit shown in Figure 2. This paper makes good use of a few microphones with simple structure to capture omnidirectional sound signals. The magneto-resistive sensor measures the current declination to acquire the heading angle of the current robot, which guides the respective robot to move in the right direction. Due to the use of low power wireless sensor network technology, the three robots can not only process sound data, magnetic data and control the motor movement, but also they can exchange information with each other and with the PC. Meanwhile, we can also measure the RSSI between robots to compute their geometrical distance from a WSN, which can avoid collisions. In this situation, this contributes to accomplishing their searching objective in close collaboration with each other.

**Figure 3:**
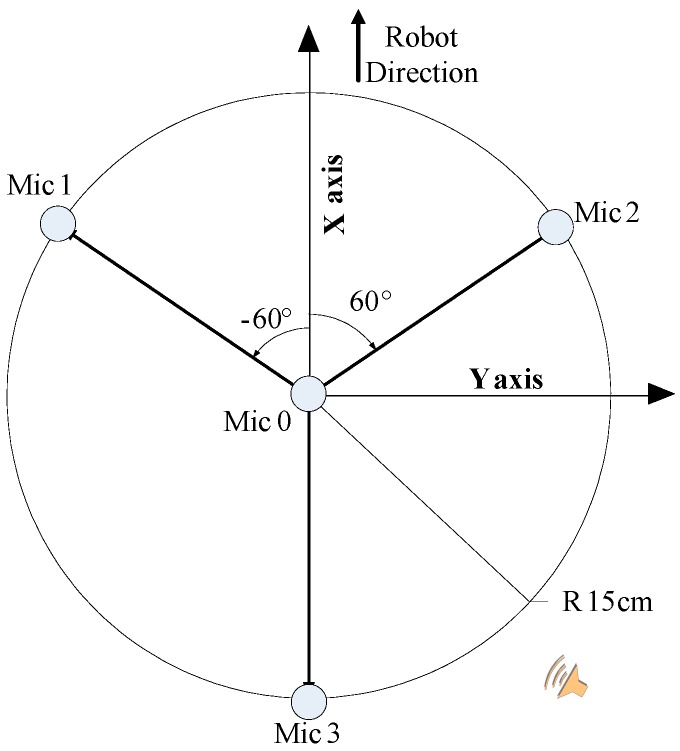
Arrangement of the 4-chnanel microphone array.

In this paper, the arrangement of the 4-channel microphone array is as shown in Figure 3. It centers on the microphone 0 and its radius is 15 cm, presenting 120° round-shaped placement. Designing such a shape assists the rough estimation of sound source location by making use of three arbitrary microphones, and the system has the ability to make a pre-judgment of sound location by the calculation of TDOA. We illustrate this situation when the sound source is in the position in Figure 3. It is not difficult for us to find out the sound direction according to the difference of time delay between microphone 2 to microphone 0 and microphone 3 to microphone 0.

The structure of the robot body mainly consists of left motor, right motor and all-direction wheel. Thanks to the two motors drive and the above structure, the robot can move forwards and turn left or right.

DSP processor acquires all sensor outputs, performs signal processing, communicates with other robots and the PC, and adjusts the speed and direction of the left and right DC motors. A pulse width modulation (PWM) technique is used to control the motors. The paper has proposed the new mapping of PWM duty ratio and the output of PID. According to the mapping, the speed and direction of robot is varied by flexibly changing a PWM duty cycle of corresponding motor.

**Figure 4:**
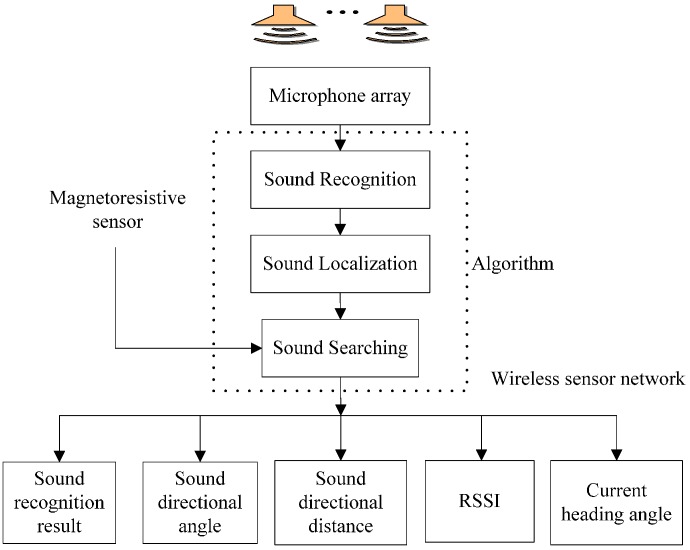
Functional architecture of the mobile robots in the WSN.

The three-robot system using WSN is implemented to accomplish the sound target-searching task, and meanwhile it can output measurement values. These parameters are sound recognition result, sound directional angle and the distance between the robot itself and the sound source, RSSI between robots and the current heading angle and they are sent to PC using the WSN. These parameters are involved in the subsequent sound signal processing and sound recognition, sound localization, and target searching algorithm. Figure 4 shows the functional architecture of the mobile robots.

## 3. Sound Signal Processing and Recognition Algorithm Based on Microphone Sensors

Sound signals are non-stationary and complex; therefore it is difficult to work out sound recognition problem, especially with pop music signals in the additional noise. This paper takes the following steps to try to solve this situation. Firstly, we choose proper sound signal as the experimental signal, and then apply the improved spectral subtraction algorithm for noise reduction according to the experimental audio signal features. Thirdly, we extract the Mel-frequency cepstral coefficient (MFCC), which has been proved to be effective and robust under various conditions. Finally, we judge the right sound signal type based on the feature template matching method.

### 3.1. Experimental Signal in Three-Robot System

Three pure sound signal segments shown in Figure 1 are selected from three songs, in which signal A is from *Daughter of the Heaver*, signal B is from *Original Dream* and signal C is from *With an Orchid.* This paper chooses music signals as experimental signals due to the fact that its sounds are not usually so strident. Meanwhile, the research also tries to verify that the speech recognition can be applied to the music signal based on the MFCC feature.

The time intervals of the experimental signals are all 10 s, in addition, we play them in turn for one sound target searching in TOC. In order to avoid the misunderstanding of recognition errors in the junctions between playing signals A and B (or B and C, C and A) i.e., when the last part of signal A is collected by the microphone array, and it could output the right signal type A after processing the acquired signal, but perhaps signal B is playing at this time, so we use a white noise signal to link them. When the robot recognizes the current signal as *other*, not A, B, and C according to the following sound recognition method, it will not move forwards. Meanwhile, we add the rated white noise (the signal to noise ratio (SNR) is about 16 dB using Equation (7)) to the three pure signals to illustrate validity of the sound recognition algorithm. To some extent, in this way this simulates the real environment. Thus, it implies that three pure sound signal is corrupted by the additional noise.

Three noisy signals A, B, C and the white noise are all played at 44.1 KHz, and the system is sampled at 32 KHz to localize the sound source. The sample interval is controlled by timers on the DSP. Although the interval is only 2 s, it produces a large amount of data. Thus, on the one hand this paper meets the Shannon Sampling Theorem, on the other we have lessened the computational burden in the DSP by down-sampling at 8 KHz during the sound recognition.

### 3.2. Improved Spectral Subtraction (ISS) for Sound Signal Enhancement

Compared with [21], an improved method is used in the research, which has achieved effective noise removal results. Furthermore, it can be widely used due to its simplicity and it is easy to implement. Therefore, it benefits the real-time function of the system, which is different from [22,23],The detail of the improved spectral subtraction procedure is shown as follows:

*Step 1*: Reducing sampling frequency of both noisy sound signal *S*(*L*) and statistical white noise signal *W*(*L*) from 32 kHz to 8 kHz, and preprocessing the signals by using the expressions:
  (1) $$\tilde{X}(n)=S[(k\times m+1):(k\times m+n)]\times w\left[n\right]\;0\leq k\leq K-1$$
  (2) $$\tilde{N}(n)=W[(k\times m+1):(k\times m+n)]\times w\left[n\right]\;0\leq k\leq K-1$$
  where *K* is the number of audio signal frame, *m* (*m* = 400) is the frame-to-frame overlap, *w*[*n*] is the *Hanning* time window, and *n* (*n* = 1024) is the length of the window *w*[*n*].
*Step 2*: Evaluating $\tilde{X}(n)$ spectral using Fast Fourier Transform (FFT) and saving the current phase angle $\tilde{\omega }(n)$.
*Step 3*: Calculating $\tilde{N}(n)$ spectral using FFT and averaged estimation μ(n) by using:
  (3) $$\mu (n)=E\{\left|FFT(\tilde{N}(n))\right|\}=\frac{1}{K}\sum_{i=1}^{K}\left|FFT(\tilde{N}(n))\right|$$
*Step 4*: Comparing their power spectra and reducing noise in an improved way by using:
  (4) $$\psi ={\left|FFT(\tilde{X}(n))\right|}^{2}-\mu {\left(n\right)}^{2}$$
  (5) $$\left|\tilde{S}(n)\right|=\left\{ \begin{array}{l} \;\;\alpha \times \left|FFT(\tilde{X}(n))\right|\;\;\;\\ \sqrt{{\left|FFT(\tilde{X}(n))\right|}^{2}-\beta \mu {\left(n\right)}^{2}} \end{array}\right.\begin{array}{l} \psi \leq 0\\ \psi >0 \end{array}$$
  where *α* is the adjusting factor, *β* is the excessive factor, and they can change with SNR.
*Step 5*: Evaluating the pure sound signal $\tilde{S}\left(L\right)$ by using:
  (6) $$\tilde{S}\left(L\right)=\sum_{i=L}\left(real(IFFT(\left|\tilde{S}(n)\right|e^{\tilde{\omega }(n)})\right))$$
  where in *L* is the length of the sampling audio signal.

The objective evaluation of noise reduction is the error size between the input and the output signal. SNR is the conventional method to evaluate the effect of noise reduction.

Assume that y(n) is the noisy audio signal, s(n) is the pure signal and $\hat{s}(n)$ is the audio signal after noise reduction. So the input and output SNR are defined respectively as:

(7) $$SNR_{in}=10\mathrm{lg}\frac{\sum_{n=0}^{L}s^{2}(n)}{\sum_{n=0}^{L}{\left[y(n)-s(n)\right]}^{2}}$$

(8) $$SNR_{out}=10\mathrm{lg}\frac{\sum_{n=0}^{L}s^{2}(n)}{\sum_{n=0}^{L}{\left[\hat{s}(n)-s(n)\right]}^{2}}$$

**Figure 5:**
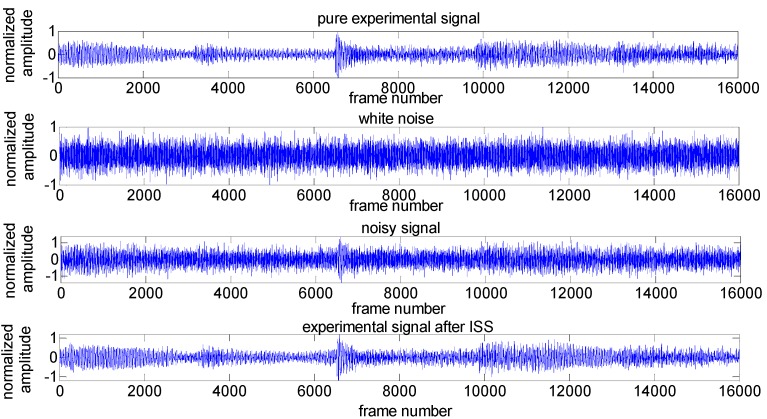
Results of experimental signal after ISS with SNR = 0 dB.

Figure 5 shows the evaluation of noise removal results in the 2-s experimental signal by using the improved spectral subtraction at SNR level of 0 dB, where *α* = 0.01, *β* = 2.1 [24]. The comparative results of the output SNR in this experiment is 6.1549 dB.

### 3.3. Mel-Frequency Cepstral Coefficients (MFCCs) Extraction

According to human auditory characteristics, the relation of Mel-frequency and real frequency can be described by Equation (9) wherein *f* (Hz) is the real frequency [25]:

(9) $$Mel(f)=2595\times \log(1+\frac{f}{700})$$

Audio signals have been traditionally characterized by MFCCs or some other time–frequency representations such as the short-time Fourier transform. The key feature of MFCC extraction algorithm is the frequency standard transform and it is changed from linear frequency to Mel-frequency scale, which coincides with human auditory characteristics. The details of the traditional MFCCs computation procedure are as follows:

*Step 1*: Preprocessing the estimated pure signal $\tilde{S}\left(L\right)$ by using:
  (10) $$X(n)=\tilde{S}[(k\times m+1):(k\times m+n)]\times w\left[n\right]\;0\leq k\leq K-1$$
*Step 2*: Calculating power spectra of Y(*n*) by using:
  (11) $${\left|Y_{a}(n)\right|}^{2}{=\;|\mathrm{FFT}(X(n))|}^{2}$$
*Step 3*: Computing the Mel-frequency bank energies output M_Y(p) by using:
  (12) $$M\_Y(p)=\sum_{n=0}^{N-1}|Y_{a}(n){|}^{2}H_{p}(n)\;0\leq p\leq P-1$$
  (13) $$H_{p}(n)=\left\{ \begin{array}{l} \;0\;\;\;\;\;\;\;\;\;\;n<f(p-1)\\ \;\frac{n-f(p-1)}{f(p)-f(p-1)}\;\;\;\;f(p-1)<n<f(p)\\ \;\frac{f(p+1)-n}{f(p+1)-f(p)}\;\;\;\;f(p)<n<f(p+1)\\ \;0\;\;\;\;\;\;\;\;\;\;n>f(p+1) \end{array}\right.$$
  where *H_p_*(*n*) is the triangular Mel-filter banks, *P* is the number of filter bank, and *f*(*p*) is the central frequency of the *p*th Mel-filter.
*Step 4*: Implementing logarithmic transform by using:
  (14) $$S(p)=Log\left(\sum_{n=0}^{N-1}|Y_{a}(n){|}^{2}H_{p}(n)\right)\;0\leq p\leq P-1$$
*Step 5*: Implementing discrete cosine transform (DCT) using:
  (15) $$C(q)=\sum_{p=0}^{P-1}S(p)\cos(\pi q(p-0.5)/P)\;0\leq q\leq P-1$$

The experimental results show that the first 3–16 cepstral coefficients are used effectively when there are 24 cepstral coefficients. Meanwhile, we regard these 14-dimensional MFCC vectors as the sound feature template to identify the different sound signal type.

### 3.4. Template Matching Based on KNN

The template matching method has been used extensively in pattern analysis applications [26,27], and maximum likelihood is its classification criterion. A significant reduction in the computational complexity is achieved by an important special case in experiments.

We have investigated the K-nearest neighbor (KNN) classification method. KNN is a simple supervised learning algorithm where a new query is classified based on the majority class of its nearest neighbors. A commonly used distance measure is the Euclidean distance by using:

(16) $$d(x,y)=\sqrt{\sum_{i=1}^{P}{\left({\hat{C}}_{i}-C_{i}\right)}^{2}}$$

wherein the $\hat{C}$ is the feature vectors extracted at the testing stage and C is the feature template vectors extracted at the training stage.

**Figure 6:**
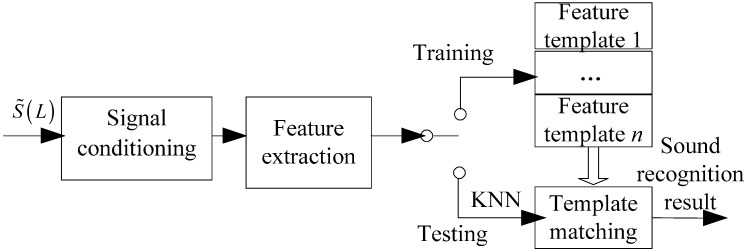
Functional architecture of template matching.

Figure 6 shows the architecture of template matching. In our experiments, we have utilized different sound signal features for training and testing sets. At the training stage, we extract the MFCCs of three pure signals and then they are stored into a template database denoted template A, B, C, while at testing stage, we collect MFCCs of three noisy signals and then compare them with template database based on the KNN classification algorithm.

For example, the current signal is type A, each robot can compute its likelihood among template A, B, C by Equation (16), and we find out that maximum likelihood appears between the current signal and template A. Consequently, the result of sound recognition is type A. It should be mentioned that once all the maximum likelihoods between the current signal and the template database are lower than a certain threshold, the robot will consider the current signal type as *other*, i.e., not the type A, B, and C. Similarly, three robots will obtain their respective results and send them to the PC using the WSN and the PC makes the voting judgment. The whole process embodies their collaboration in this way.

## 4. Sound Localization and Searching Algorithm Based on Microphone Array and Magneto-Resistive Sensor

The sound localization algorithm includes estimation of TDOA based on a microphone array and geometrical localization. Firstly, compared with [28], we confirm that it is necessary to estimate TDOA by making use of the improved generalized cross correlation (IGCC) method. Then source localization employs a spherical interpolation method to fix the sound position, and it also combines the geometrical position of the microphone array and the magnetic declination measured by the magneto-resistive sensor. Finally, we have proposed a new mapping of heading angle deviation and PWM duty ratio, and the deviation is the output of PID closed-loop control algorithm. Thus, such mapping can direct the motor to move in the right direction smoothly.

TDOA between two direct signal paths from different microphones has the advantages of requiring a lesser number of microphones and it is easily realized with a small amount of calculation, which is very important in a DSP system.

### 4.1. TDOA Based on Microphone Array

According to the arrangement of the 4-channel microphone array above, the system is sampled at 32 KHz to gain the high estimation accuracy of TDOA. In the experiment, we discover a problem that synchronization noise is caused by the hardware circuits of the microphone array, which could produce fake peak values in traditional GCC. In this situation, the estimation of TDOA is wrong, therefore we design the FIR band-pass filters (band-pass frequency is from 200 Hz to 8 KHz) to improve the traditional GCC method, which can avoid such an error to some extent. However, it is not enough to design such a filter under spectral overlapping conditions between the audio signals and environmental noise (or digital circuit components). Therefore, superposition of cross power spectral between noise and the useful audio signal is also used to improve the TDOA estimation precision.

**Figure 7:**
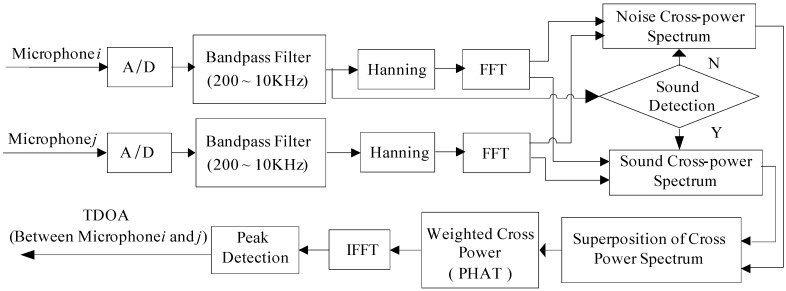
Architecture of TDOA by IGCC.

The research uses a 4-channel microphone array, and this paper needs ${C_{4}}^{2}\;=\;6$ groups TDOA between two arbitrary microphones. Figure 7 shows the architecture of estimating the time delay by the IGCC method and it is illustrated for microphone *i* and microphone *j*. Microphone *i* is used to detect whether the collected signal is a sound signal based on short time average zero-passed rate, which can remove the passive effect of ambient noise. If the current audio signal frame is noise caused by the digital circuits or environmental noise, its power spectra is denoted as Noise Spectra. Afterward, if the following frame is a noisy audio signal, its power spectra is denoted as Noisy Audio Signal Spectra. Therefore, the applicable power spectral computing TDOA is their difference between noisy audio signal spectral and noise spectra. 

The audio signal echoes and environmental noise leads to unobvious cross correlation peak values between microphone signals, which can reduce the accuracy of the corresponding TDOA. To avoid this situation, we implement the Phase Transform (PHAT) by using the weighting $\psi_{p}(f)$ [29] wherein the $G_{x_{1}x_{2}}(f)$ is the cross-power spectrum between microphone signals xl and x2:

(17) $$\psi_{p}(f)=\frac{1}{\left|G_{x_{1}x_{2}}(f)\right|}$$

Then, we adopt Inverse Fast Fourier Transform (IFFT) and find its peak value. Finally, the time *τ_ij_* of the peak in IGCC represents the time delay estimation.

**Figure 8:**
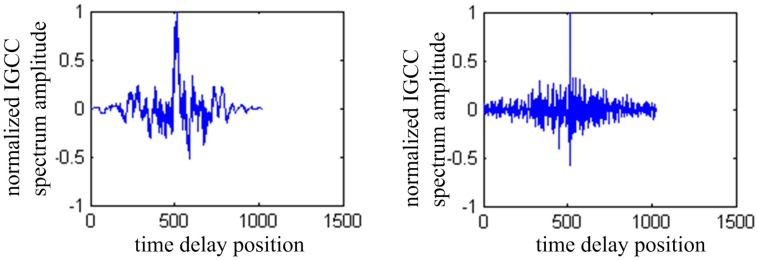
TDOA result of traditional GCC and IGCC.

Figure 8 shows the TDOA result of traditional GCC and IGCC. In the left figure, the moment of the peak is not sharp when using GCC, whereas the IGCC has a sharper peak shown in the right one.

### 4.2. Source Localization Based on the Spherical-Interpolation Method

Differences in arrival time of sound signals among the microphone array are measurable in the above case, and they can be used to fix the location of a certain source. Under the conditions of the least-squares formula, the basic idea of the spherical interpolation method [30] is to solve a set of equations based on TDOA and geometric relations of the microphone array.

**Figure 9:**
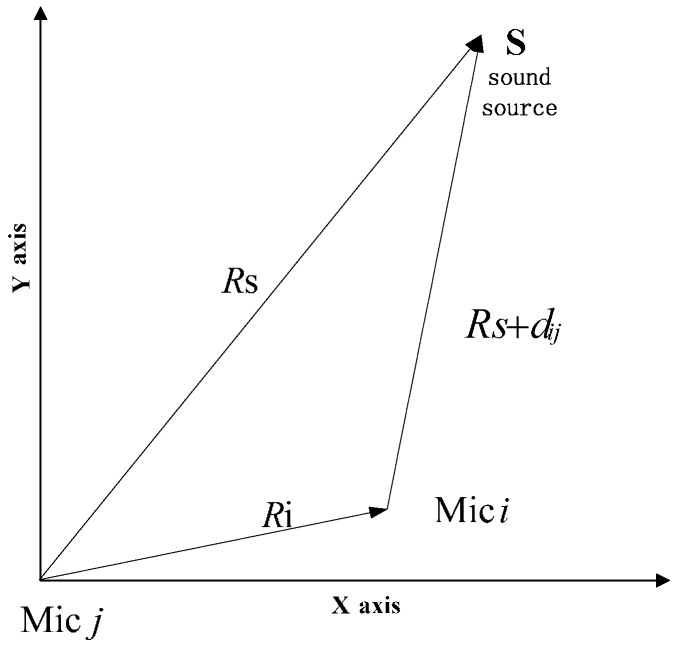
Diagram illustrating geometric relations among *m_i_*, *m_j_* and **S**.

We first map the spatial origin into two arbitrary microphone sensors, say the *i*th and *j*th as shown in Figure 9. The distance from the *j*th microphone to the *i*th microphone is denoted *R_i_* = |***r**_i_*|. The distance between the sound source and *j*th microphone is denoted *R_s_* = |***r**_s_*|, and the distance between the source and *i*th microphone is denoted *R_s_* + *d_ij_*, where ***r**_i_* and ***r**_s_* are the corresponding vectord, and *d_ij_* is distance that is calculated based on TDOA between *i*th and *j*th microphone. These quantities appear in Figure 9.

We have the following basic relations based on the Pythagorean Theorem:

(18) $${\left(R_{s}+d_{ij}\right)}^{2}={R_{i}}^{2}-2{r_{i}}^{T}r_{s}+{R_{S}}^{2}$$

(19) $$d_{ij}=\tau_{ij}\times v$$

where *v* represents the speed of the sound in air, and its value is about 342 m/s, and:

(20) $$0={R_{i}}^{2}-{d_{ij}}^{2}-2R_{s}d_{ij}-2{r_{i}}^{T}r_{s}$$

As the TDOA are typically not measured precisely, there exists a so-called “equation error” on the left hand side of Equation (20), and minimizing it in a least-squares sense providea an estimate of the true solution. Then Equation (20) becomes:

(21) $$\varepsilon ={R_{i}}^{2}-{d_{ij}}^{2}-2R_{s}d_{ij}-2{r_{i}}^{T}r_{s}$$

Assume that the number of microphones is *m*, which is denoted Mic_0_, Mic_1_,…,Mic*_m−1_*. Therefore, it is not difficult for us to solve the corresponding *m* − 1 sets of equations about the distance from the other (Mic_1_,Mic_2_,…,Mic*_m−1_*) microphones to the Mic*_0_*, and then Equation (21) becomes:

(22) $$\varepsilon_{i}={R_{i}}^{2}-{d_{i0}}^{2}-2R_{s}d_{i0}-2{r_{i}}^{T}r_{s}\;\;i=1,2,\cdots ,m-1$$

Then, the above Equations (22) can be written in matrix notation as:

(23) $$\varepsilon =\delta -2R_{s}d-2Sr_{s}$$

wherein:

$$\delta ={R_{i}}^{2}-{d_{i0}}^{2}=\left[\begin{array}{c} {R_{1}}^{2}-{d_{10}}^{2}\\ {R_{2}}^{2}-{d_{20}}^{2}\\ \vdots \\ {R_{m-1}}^{2}-{d_{(m-1)0}}^{2} \end{array}\right],d=\left[\begin{array}{c} d_{10}\\ d_{20}\\ \vdots \\ d_{(m-1)0} \end{array}\right]，S=\left[\begin{array}{c} x_{1}\;y_{1}\;z_{1}\\ x_{2}\;y_{2}\;z_{2}\\ \vdots \\ x_{m-1}\;y_{m-1}\;z_{m-1} \end{array}\right]$$

It is a fact that the equation error vector Equation (23) is linear in $r_{s}$, given $R_{S}$, and it is also linear in $R_{S}$, given $r_{s}$. The formal least-squares solution for $r_{s}$, given S, and:

(24) $$r_{s}=\frac{1}{2}{S^{*}}_{w}(\delta -2R_{S}d)$$

wherein:

$${S_{w}}^{*}={\left(S^{T}S\right)}^{-1}S^{T}$$

In the above case, when formal least-squares formula is met, sound source position ${\hat{r}}_{0}$ can be approximately estimated.

Similarly, ${\hat{r}}_{1}$,…,${\hat{r}}_{m-1}$ can be achieved. Then all the estimated coordinates ${\hat{r}}_{0}$, ${\hat{r}}_{1}$,...,${\hat{r}}_{m-1}$ are converted into reference coordinates whose center is Mic_0_. Finally, in order to improve the accuracy of the sound position estimation, we compute their average value $\hat{r}$ by using:

(25) $$\hat{r}=\frac{1}{m}\sum_{n=0}^{m-1}{\hat{\;r}}_{n}$$

To improve the localization accuracy further, we also adopt the average estimation $\hat{r}$ of the multi-frame audio signal in the experiment.

Until now, the direction angle and the distance between the current robot and the sound source target can be computed by using the above method. Besides, we also measure the RSSI between robots to avoid their collision. The RSSI from a WSN will degrade with the increase of the geometrical distance, and the relation of the RSSI and distance between robots can be described as follows:

(26) $$R=R_{0}-10n\log(\frac{d}{d_{0}})+\xi$$

where *n* is the signal propagation constant and its range is from 3.25 to 4.5 *d* is the distance from sender. ξ is the random noise, its mean is 0 and standard difference ranges from 4 to 10. *R*_0_ is the RSSI from the distance *d*_0_, which is used for calibration.

### 4.3. Sound Searching Algorithm Based on the New Mapping of Heading Angle Deviation output of PID and PWM Duty Ratio

At the last stage, we have estimated the direction angle of the sound source (denoted as $\theta_{s}$) and the distance between the current robot and sound source (denoted as *d_rs_*). A magneto-resistive sensor can measure the current magnetic declination as the current heading angle of the robot (denoted as $\theta_{h}$). Therefore, we regard the sum of current heading angle of robot and the direction angle of the sound source as the target heading angle of searching sound source (denoted as $\theta_{t}=\theta_{s}+\theta_{h}$).

As the current heading angle of the robot, variable $\theta_{h}$ will have different values in different positions, thus, we redefine the new ${\tilde{\theta }}_{h}$ instead of $\theta_{h}$. In this paper, we consider $\theta_{t}$ and ${\tilde{\theta }}_{h}$ as the input of a PID closed-loop control algorithm, and then we can obtain the output of PID, i.e., the heading angle deviation (denoted as $\theta_{d}=\theta_{t}-{\tilde{\theta }}_{h}$). We control the two motors effectively according to the new mapping of the heading angle deviation $\theta_{d}$ and the PWM duty ratio described as follows.

**Figure 10:**
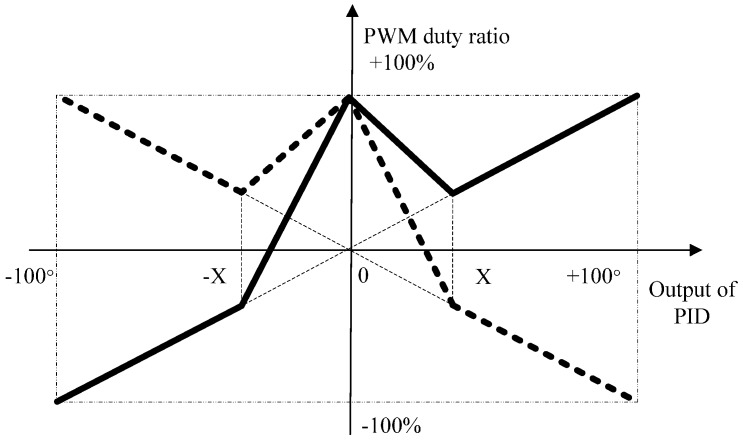
New mapping of heading angle deviation and PWM duty ratio.

Figure 10 shows the mapping relation between them. The solid line represents the PWM duty ratio of one motor, while the dashed line represents the other. The threshold value X is selected in the experiment, and heading angle deviation $\theta_{d}$ is limited to ±100°.

According to the above mapping relation, the PWM duty ratios of the two motors have different directions (one is positive and the other is negative), meanwhile their absolute values are relatively high when the absolute value output of the PID is very big. In this situation, the robot can adjust direction rapidly. When the absolute value output of the PID is very small, their PWM absolute duty ratios are also high, but they have the same direction, which guides the robot to move forward rapidly. As for the positive or negative heading angle deviation $\theta_{d}$, it mainly controls the robot to turn right or left. It is important that the PWM duty ratio is continuous in the mapping, which benefits to the stability of motions. The sound searching algorithm proposed in this paper can adjust the direction and speed flexibly and rapidly in order to accomplish the task smoothly. To avoid the detrimental effects of external intense magnetic fields and temperature, we use the mean value of the magneto-resistive sensor ten-time measurement results ${\tilde{\theta }}_{h}$ as the current robot heading angle.

## 5. Design and Implementation of a Sound Target-Searching Three-Robot System in a WSN

**Figure 11:**
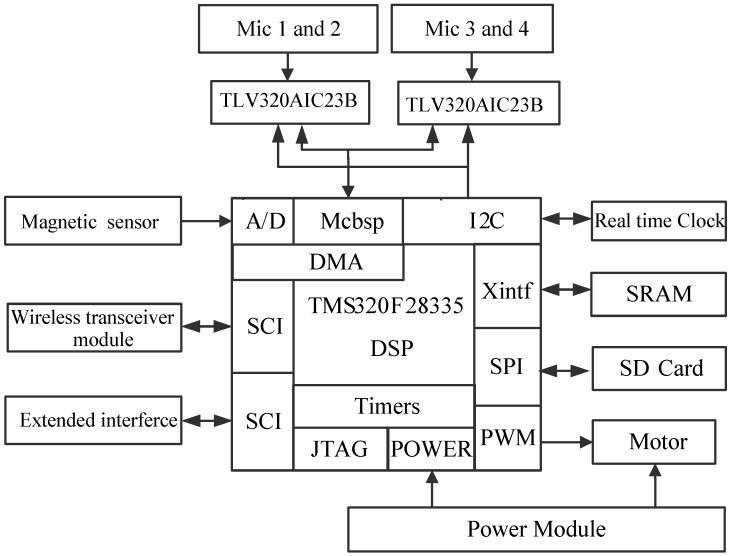
Schematic architecture of DSP system.

In order to verify the above algorithms, a real-time sound target-searching three-robot platform in a WSN is implemented using the high-performance, floating point DSP TMS320F28335 of Texas Instruments (TI, Dallas, TX, USA), which combines the advantages of providing rich controlling functions with the capacity of implementing complex algorithms. It acquires the output signal of the 4-channel microphone array and magneto-resistive sensor synchronously by the direct memory access (DMA) transfer technology, which achieves the data transfers efficiently, especially for the massive audio data collection. After signal conditioning, the DSP processor completes the sound recognition and localization, and sound searching algorithm. Then their processed results are output to the PC through the WSN. The result information mostly includes the sound identification result, sound source direction angle $\theta_{s}$ and current heading angle of the robot ${\tilde{\theta }}_{h}$, and the RSSI between robots. Finally, the PC makes a judgment and returns the decision result to the right robot, in addition, the right robot starts to move in the right direction while the other robots keep still. Figure 11 shows the schematic architecture of the DSP system.

The output signals of the four channel sound sensors are amplified by fixed-gain microphone amplifiers MAX9812 and then output to the ideal high-performance stereo audio codec TLV320AIC23B of TI. It integrates analog functionality, and supports data-transfer word lengths of 16, 20, 24, and 32 bits, meanwhile its sample rates range from 8 kHz to 96 kHz. Then it is encoded and transferred into DSP through a Multi-Channel Buffer Serial Port (Mcbsp).

The 2-axis magneto-resistive sensor HMC1022 measuring circuit is designed to obtain magnetic sensing signals which are then amplified by AD623instrumentation amplifiers to obtain the current heading angle ${\tilde{\theta }}_{h}$, which can help the robot search target.

The Wireless Communication Unit selects the CC2430, a true System-on-Chip (SoC) solution specifically tailored for IEEE 802.15.4. Furthermore, it combines the excellent performance of the leading CC2420 RF transceiver with an industry-standard enhanced 8051 MCU and it also has a built-in RSSI giving a digital value. It can easily communicate with the DSP through a Serial Communications Interface (SCI). Its function is to help us exchange information between the robots and the sink node.

The L293D is designed to drive the two DC motors and it provides bidirectional drive currents up to 1 A at voltages from 4.5 V to 36 V. The high density mounting phototransistor optically coupled isolators TLP521 are applied to separate the signal control circuit from the motor drive circuit in order to suppress the electro-magnetic interference (EMI). Based on the motor drive circuit, the General Purpose Input/Output (GPIO) on the DSP can control the movement direction of the motors and the PWM module could adjust the speed of the motors. 

To train the audio feature template, we expand the SD Card through a Serial Peripheral Interface (SPI) on the DSP, meanwhile we create a FAT16 file system, which has access to 2 GB and can store the massive data effectively. At the time of creating the file system, we use the current time information as the file name to avoid repeat denominations, which can be achieved by a real time clock/calendar measuring circuit. The X1226 can provide the current precise time information and it is connected with the DSP through an Inter-Integrated Circuit (I^2^C). Moreover, due to the massive audio data, we also expand to 512 K × 16 high-speed asynchronous Static RAM (SRAM) IS61LV51216.

## 6. Experiment and Results

### 6.1. Experimental Setup

**Figure 12:**
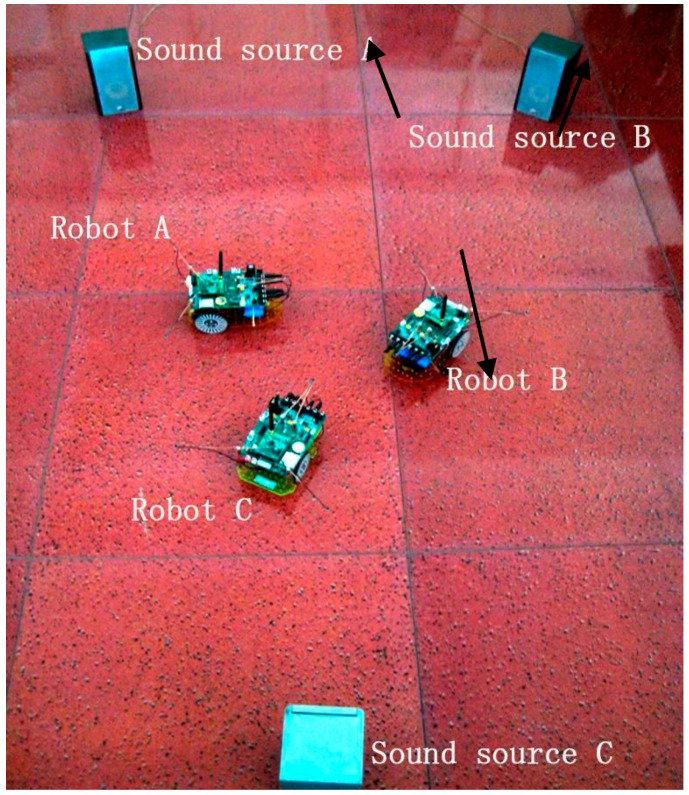
Experimental process.

The experiments were conducted in a room shown in Figure 12. The experimental area spanned 6 m × 8 m and it was surrounded by walls, desks and chairs. The whole experimental process is done in a laboratory environment.

The experimental process can be described as follows:

Firstly, place three sound boxes A, B, and C in different positions in the room; they correspond to the three noisy sound signals A, B, and C described above. Play three music signal segments and white noise by turns.

Secondly, each robot collects and processes its audio signal data; they acquire their respective results of sound identification, sound source position, RSSI and the heading angle of the robot. Then they send them to the PC through the WSN.

**Figure 13:**
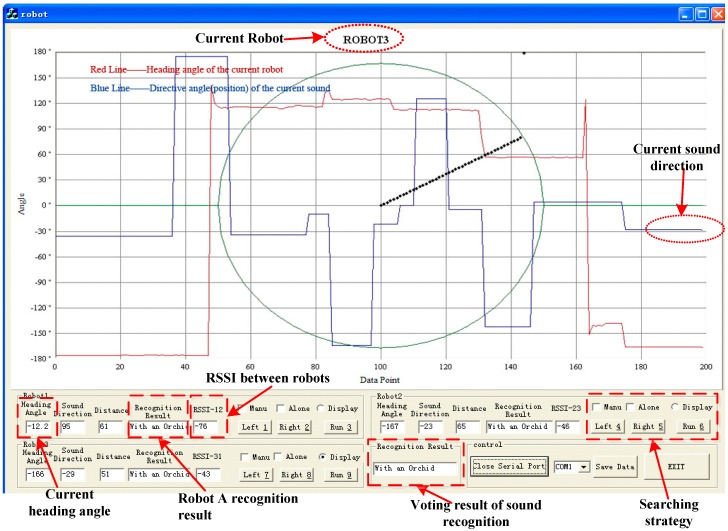
PC interface.

Thirdly, the PC displays the above information from each robot shown in Figure 13; meanwhile it has the ultimate power to make a comprehensive judgment and then return the decision result to the right robot by using the WSN. For example, when the received sound recognition result sent by the three robots is type A, A and B or type A, A, and C, the PC may consider it as type A.

Finally, the right robot, i.e., the robot denoted A in the above example, moves to the sound box A in the following 3 s while robots B and C stay still. By the same strategy, the three robots accomplish the sound source target searching task cooperatively.

### 6.2. Sound Recognition Result

To verify the above sound recognition algorithm, the feature template parameters are first extracted at the training stage, and then their validity is tested by multiple experiments. The results show that sound identification accuracy of each robot alone can be higher than 80% in ten experiments, and it can be increased into 90% in a cooperative way. Detailed results are shown in Table 1 in which the signal type C is played and *other* represents that all the likelihoodz are under a certain threshold.

In Table 1, the sound recognition result is sometimes wrong under intensive background noise, and the decrease of sound identification accuracy at this time was caused by the following reasons: firstly, the experimental sound signal itself is quite complex, and featured by variety and diversity. The sound recognition of such a signal itself is difficult. Then, the interval of the matched template at the training stage is 2 s, which is only a little part of the whole 10 s experimental signal and the features are not covered completely. This type of sound identification is different from traditional isolated words.

The interference factors of the above background noise mainly includes artificially added white noise with SNR higher 18 dB, echoes, noise from motors and other noises. Although sometimes the result of sound recognition is wrong under intensive noise conditions, the three-robot system can improve the accuracy to some extent, and accomplish the target searching task cooperatively in the WSN. Besides this paper, the recognition experiments of music signals A and B are also done, and the results are consistent.

### 6.3. Sound Localization Results

The above sound localization algorithm is employed to fix the current position of the sound source. The experimental signal plays at 44.1 kHz and the sampling rate is 32 kHz. The distance between the three robots and three sound boxes is about 0.1~3 m, and the range of sound detection is –180°~+180°. In the laboratory environment, Table 2 illustrates the sound localization results under 12 working conditions and 25 experiments have been done for each condition.

The results show that the angle error of sound localization is lower than ±10°, but the distance accuracy is not so high. Therefore, the robot mainly depends on the angle information to implement sound localization. The distance has also been computed to judge whether the right robot arrives at the right target. In the experiment, the distance threshold is set at 20 cm, i.e., when the test distance is shorter than the threshold; the robot will stop moving forwards and accomplish its task.

To avoid collisions between robots, we use RSSI to measure their rough geometrical distance. The RSSI and the distance from sender have certain relation by using Equation (26). Thus, once the RSSI is measured, the test distance is known. Table 3 illustrates the RSSI localization results under 12 working conditions and 25 experiments have been done in each condition. The results show that the RSSI accuracy changes with their distance between robots. When their distance is within 120 cm, the accuracy is relatively high, otherwise, it becomes low. Therefore, we generally use the RSSI to judge the rough short distance between robots in order to avoid their collision. In this way, their collaboration is meaningful.

In the experiment, the distance threshold for collision avoidance is set at 30 cm (the RSSI distance accuracy is about 3 cm) according to the measured RSSI between robots, i.e., when the test distance is shorter than the threshold, the robots will adjust themselves automatically in order to keep away from each other. The sound localization and RSSI results can be also seen from Figure 13.

### 6.4. Sound Searching Result

After accomplishing the sound recognition and localization in TOC, the searching function will be realized in the WSN according to the current position of both the right robot and the sound target. The communication bandwidth of the WSN is lower than 128 K and the accuracy of the current heading angle measured by magneto-resistive sensor is under 2°, which assists the robots to search for and find the source target.

According to the proposed new mapping, once the target heading angle $\theta_{t}$ is known, the robot can adjust its speed and direction automatically and flexibly. Whenever each robot has completed its searching function in turn, it moves forward about 30 cm. In the experimental room (the diameter is no larger than 6 m), this can be done 10 times at most in the whole searching process and three robots arrive at the three sound boxes, respectively.

As Figure 13 shows, apart from the cooperative searching method based on PC, the searching strategy also includes a respective searching method for its target alone without the help of other robots and the PC, and manual control searching for the right target. In the experiment, we can also move the sound boxes, and the right robot will perform the same function of searching the right sound target as before.

### 6.5. Time Consumption Evaluation of Algorithms

Time consumption is evaluated using the profile tool provided by Code Composer Studio (CCS). Time expenditure of the audio signal collection is 2 s, controlled by the timer on the DSP. The average time consumed in sound recognition, sound localization and the sound searching algorithm is about 3 s. Then the motion of robot needs the following 3 s. To sum up, the robot consumes approximately 8 s to accomplish each searching process. The system leads to the above conclusion based on a number of experiments under different circumstances. The results show that a sound target-searching three-robot system in a WSN can implement the function of sound identification and localization, and sound source searching. The system obtains good results with preliminary intelligence.

## 7. Conclusions

In this paper, a new sound target-searching three-robot system based on a 4-channel microphone array and a magneto-resistive sensor in a WSN is presented. The structural design of the system, sound recognition and localization method, sound searching method according to the new mapping, and hardware platform based on DSP are described in detail. The experimental results show that: (1) The designed three-robot system accomplishes the sound target-searching function smoothly in a WSN. It can output some parameters, including the type of sound signal, position of the current sound source, the RSSI between two robots to avoid collisions, and the current heading angle of each robot; (2) A PC displays the above parameters, and exchanges them with the three robots. It also has the ultimate power to make comprehensive judgments, and returns the decision result to the right robot in the WSN. When the system works under background noise, it can also improve the accuracy of sound identification; (3) Due to TOC, the structure of the system is relatively simple and the accuracy of cooperative sound identification can rise to 90% in contrast to 80% of each robot alone. The angle error of sound localization is lower than ±10°, and current heading angle measurement lower than 2°. This system integrates the application of multiple-sensor technology, pattern recognition, wireless sensor networks and robot technology.

## Figures and Tables

**Table 1:** Sound recognition results of signal type C.

Number	Robot A	Robot B	Robot C	PC
1	C	C	C	C
2	C	C	C	C
3	C	C	C	C
4	*Other*	C	C	C
5	C	C	C	C
6	*Other*	*Other*	C	*Other*
7	C	C	C	C
8	C	C	C	C
9	C	*Other*	C	C
10	C	C	C	C

**Table 2:** Sound localization results.

Number	Average of Test Angle (°)	Standard Deviation of Test Angle (°)	Average of Test Distance (cm)	Standard Deviation of Test Distance (cm)	Real Angle (°)	Real Distance (cm)
1	27.5	4.1	17.2	2.2	30	20
2	28.2	3.2	88.3	3.6	30	100
3	26.6	4.9	228.5	4.0	30	200
4	33.1	4.5	316.1	4.8	30	250
5	−56.5	4.9	30.4	4.5	−60	40
6	−60.6	3.7	128.7	5.1	−60	150
7	−58.3	3.9	240.1	4.9	−60	200
8	−61.7	3.2	361.2	4.7	−60	300
9	117.1	4.5	37.6	4.3	120	50
10	123.8	4.9	65.7	4.6	120	80
11	122.4	3.3	158.1	5.6	120	100
12	121.2	3.4	135.3	5.1	120	120

**Table 3:** RSSI localization results.

Number	Real Distance (cm)	RSSI (dBm)	Average of Test Distance (cm)	Standard Deviation of Test Distance (cm)
1	0	−10	3.5	2.2
2	20	−30	22.5	2.3
3	40	−39	41.5	2.1
4	60	−45	59.1	2.6
5	80	−53	97.6	3.6
6	100	−54	110.2	3.1
7	120	−56	126.7	4.0
8	140	−56	128.2	4.2
9	160	−60	168.1	3.8
10	180	−58	148.7	5.1
11	200	−66	249.1	4.8
12	220	−65	234.5	4.9

## References

[1] Turaga P., Ivanov Y. (2011). Diamond sentry: Integrating sensors and cameras for real-time monitoring of indoor spaces. IEEE Sens. J..

[2] Wang X., Wang S., Bi D. (2009). Compacted probabilistic visual target classification with committee decision in wireless multimedia sensor networks. IEEE Sens. J..

[3] Drummond T., Cipolla R. (2002). Real-time visual searching of complex structures. IEEE Trans. Pattern Anal. Mach. Intell..

[4] Kelly R. (1996). Robust asymptotically stable visual serving of planar robots. IEEE Trans. Robot. Autom..

[5] Flanagan J.L., Berkley D.A., Elko G.W. (1991). Auto-directive microphone systems. Autod. Micropho. Syst..

[6] Zheng Y.R., Goubran R.A., Tanany M.E. (2005). A microphone array system for multimedia applications with near-field signal targets. IEEE Sens. J..

[7] Tamai Y., Kagami S., Mizoguchi H. Real-time 2 dimensional sound source localization by 128-channel huge microphone array. Proceedings of the 13th IEEE International Workshop on Robot and Human Interactive Communication.

[8] Yamamoto S., Nakadai K., Nakano M. Real-time robot audition system that recognizes simultaneous speech in the real world. Proceedings of the 2006 IEEE/RSJ International Conference on Intelligent Robots Systems.

[9] Dowling E., Linebarger D., Tong Y. (1992). An adaptive microphone array processing system. Microproc. Microsyst..

[10] Desloge J.G., Rabinowitz W.M., Zurek P.M. (1997). Microphone-array hearing aids with binaural output. I. Fixed-processing systems. IEEE Trans. Speech Audio Process..

[11] Compernolle D.V., Ma W.Y., Xie F. (1990). Speech recognition in noisy environments with the aid of microphone arrays. Speech Commun. J..

[12] Nguyen D.C., Shen G.H., Jung H.Y. Performance improvement of speech recognition system using microphone array. Proceedings of the 2008 IEEE International Conference on Research, Innovation and Vision for the Future.

[13] Schewea H., Schelter W. (1997). Industrial applications of magnetoresistive sensors. Sens. Actuators A Phys..

[14] He W.H., Yan G.Z., Guo X.D. The application of magnetoresistive sensor in detecting the capsule’s localization in GI. Proceedings of the 2006 International Conference on Communications, Circuits and Systems Proceedings.

[15] Ahn H.S. (2009). Vision-based magnetic heading sensor for mobile robot guidance. Electron. Lett..

[16] Chen G.Z., Shen C.F., Zhou L.J. (2009). Design and performance analysis of wireless sensor network location node system for underground mine. Min. Sci. Technol..

[17] Zhang X.H., Fang J.L., Yu X. (2010). Design and implementation of nodes based on CC2430 for the agricultural information wireless monitoring. Comput. Autom. Eng..

[18] Chen X., Yan F.L. Wireless ultrasonic data transmission based on CC2430 chip. Proceedings of the 2009 International Conference on Test and Measurement.

[19] Ishida H., Nakayama G., Nakamoto T. (2005). Controlling a gas/odor plume-searching robot based on transient responses of gas sensors. IEEE Sens. J..

[20] Grenier Y. (1993). A microphone array for car environments. Speech Commun. J..

[21] Karam M., Aglan H. Spectral subtraction of noise in speech processing applications. Proceedings of the 40th Southeastern Symposium on System Theory.

[22] Xia Y., Liang Y., Bao C.C. A modified spectral subtraction method for speech enhancement based on masking property of human auditory system. Proceedings of the 2009 International Conference on Wireless Communications & Signal Processing.

[23] Furuya K., Kataoka A. (2007). Robust speech dereverberation using multichannel blind deconvolution with spectral subtraction. IEEE Trans. Audio Speech Lang. Process..

[24] Berouti M., Schwartz R., Makhoul J. Enhancement of speech corrupted by acoustic noise. Proceedings of the IEEE International Conference on ICASSP ‘79 Acoustics Speech, and Signal Processing.

[25] Molau S., Pitz M., Schluter R. (2001). Computing mel-frequency cepstral coefficients on the power spectrum. IEEE Trans. Acoust. Speech Signal Process..

[26] Chen C., Huang W., Tan T., Chang C.C., Chang Y.J. (2015). Using K-nearest neighbor classification to diagnose abnormal lung sounds. Sensors.

[27] Kaneko T., Hori O. Feature selection for reliable tracking using template matching. Proceedings of the 2003 IEEE Computer Society Conference on Computer Vision and Pattern Recognition.

[28] Azaria M., Hertz D. (1984). Time delay estimation by generalized cross correlation methods. IEEE Trans. Acoust. Speech Signal Process..

[29] Carter G.C., Nuttall A.H., Cable P.G. (1973). The smoothed coherence transform (SCOT). IEEE Proc..

[30] Smith J., Abel J. (1998). Closed-form least-squares source location estimation from range-difference measurement. IEEE Trans. Acoust. Speech Signal Process..

